# Delivery of PLGA-Loaded Influenza Vaccine Microparticles Using Dissolving Microneedles Induces a Robust Immune Response

**DOI:** 10.3390/pharmaceutics17040510

**Published:** 2025-04-12

**Authors:** Emmanuel Adediran, Tanisha Arte, Dedeepya Pasupuleti, Sharon Vijayanand, Revanth Singh, Parth Patel, Mahek Gulani, Amarae Ferguson, Mohammad Uddin, Susu M. Zughaier, Martin J. D’Souza

**Affiliations:** 1 Center for Drug Delivery Research, Vaccine Nanotechnology Laboratory, College of Pharmacy, Mercer University, Atlanta, GA 30341, USA; emmanuel.adediran@live.mercer.edu (E.A.); tanisha.manoj.arte@live.mercer.edu (T.A.); dedeepya.pasupuleti@live.mercer.edu (D.P.); sharon.c.p.vijayanand@live.mercer.edu (S.V.); revanth.singh.sateesh@live.mercer.edu (R.S.); parth.r.patel@live.mercer.edu (P.P.); mahekanil.gulani@live.mercer.edu (M.G.); amarae.ferguson@live.mercer.edu (A.F.); uddin_mn@mercer.edu (M.U.); 2 College of Medicine, QU Health, Qatar University, Doha P.O. Box 2713, Qatar; szughaier@qu.edu.qa

**Keywords:** microneedles, PLGA, influenza, microparticles, polymer

## Abstract

**Background:** Influenza virus is one of the major respiratory virus infections that is a global health concern. Although there are already approved vaccines, most are administered via the intramuscular route, which is usually painful, leading to vaccine hesitancy. To this end, exploring the non-invasive, transdermal vaccination route using dissolving microneedles would significantly improve vaccine compliance. Research on innovative vaccine delivery systems, such as antigen-loaded PLGA microparticles, has the potential to pave the way for a broader range of vaccine candidates. **Methods:** In this proof-of-concept study, a combination of the inactivated influenza A H1N1 virus and inactivated influenza A H3N2 virus were encapsulated in a biodegradable poly (lactic-co-glycolic acid) (PLGA) polymeric matrix within microparticles, which enhanced antigen presentation. The antigen PLGA microparticles were prepared separately using a double emulsion (*w*/*o*/*w*), lyophilized, and characterized. Next, the vaccine microparticles were assessed in vitro in dendritic cells (DC 2.4) for immunogenicity. To explore pain-free transdermal vaccination, the vaccine microparticles were loaded into dissolving microneedles and administered in mice (n = 5). **Results:** Our vaccination study demonstrated that the microneedle-based vaccine elicited strong humoral responses as demonstrated by high antigen-specific IgA, IgG, IgG1, and IgG2a antibodies in serum samples and IgA in lung supernatant. Further, the vaccine also elicited a strong cellular response as evidenced by high levels of CD4+ and CD8a+ T cells in lymphoid organs such as the lymph nodes and spleen. **Conclusion:** The delivery of influenza vaccine-loaded PLGA microparticles using microneedles would be beneficial to individuals experiencing needle-phobia, as well as the geriatric and pediatric population.

## 1. Introduction

Influenza is a highly contagious respiratory infectious disease that has been a significant cause of morbidity and mortality worldwide. Every year, on average, 8 million of these cases are reported, making it a public health concern [1]. The influenza A virus exhibits high variability due to mutations in its surface glycoproteins, leading to the emergence of different strains. For example, influenza A H1N1 and H3N2 have not only caused pandemics in the past but also continue to pose a significant threat due to their pandemic potential [2,3]. Influenza virus infection spreads primarily through droplets deposited on the nasal mucosa when an infected individual coughs or sneezes [4]. Patients with this infection may experience a wide range of symptoms, from mild manifestations, such as fever, sore throat, muscle weakness, weight loss, and runny nose, to severe complications, including death in vulnerable populations, such as immunosuppressed individuals, children, and the elderly [5]. Given the pandemic potential of influenza, research into vaccines targeting such pathogens is essential for public health security. Developing effective vaccination strategies can help mitigate future outbreaks and reduce morbidity and mortality rates. At the mechanistic level, the influenza virus mediates its infection process by using Hemagglutinin, a glycoprotein expressed on the surface of the virus structure. Upon binding with the sialic acid receptor on the host cell surface, the virus is internalized by receptor-mediated endocytosis followed by a cascade of events for the replication process [2].

To address the challenges posed by influenza, vaccination has been a key strategy in controlling its spread. One widely used method for producing influenza vaccines involves Madin–Darby Canine Kidney (MDCK) cells, which support virus replication and are highly susceptible to influenza virus infection, making them an effective platform for developing cell-based influenza vaccines [6,7]. Currently, the inactivated form of these viruses is a component of the conventional flu vaccine, providing immune protection against influenza and reducing the risk of severe illness. Despite the availability of approved flu vaccines, several challenges remain. Most influenza vaccines are administered via the invasive intramuscular route, except for FluMist, which is delivered intranasally [8]. However, intramuscular vaccination requires a trained healthcare professional, and the risk of needle-stick injuries at the injection site, coupled with vaccine hesitancy among needle-phobic individuals, contribute to poor compliance [9]. Additionally, the cold chain storage requirement for vaccines continues to pose a major challenge in developing countries. Further, the Advisory Committee on Immunization Practices in the United States did not recommend the intranasal flu vaccine for the 2016–2017 influenza season due to safety concerns [10]. Ineffective vaccines often fail to induce a strong or specific immune response, limiting their protective capabilities [11]. Therefore, there is a pressing need for more effective and patient-friendly vaccination strategies to overcome these limitations and improve global influenza prevention efforts.

Interestingly, there has been an increasing body of evidence and several attributes on the use of polymeric microparticles as carriers for antigen delivery and efficacious vaccine development [12]. The first key attribute is that the microparticle size closely mimics that of foreign pathogens targeted by the immune system. As a result, they are efficiently recognized and processed by antigen-presenting cells (APCs). Microparticles within the 1–3 µm range are particularly effective, and studies have demonstrated that antigen-presenting cells can successfully uptake microparticles smaller than 5 µm [13,14]. Secondly, polymeric microparticles offer the advantages of being biodegradable and biocompatible, making them ideal for antigen delivery. Various polymers, including hyaluronic acid, chitosan, polyanhydrides, and polyorthoesters, have been successfully utilized to enhance the delivery and presentation of antigens. These materials not only ensure controlled and sustained release but also improve the stability of encapsulated antigens, thereby enhancing the immune response [15]. Along this path, our group has extensively and recently demonstrated the use of biodegradable poly (lactic-co-glycolic acid) (PLGA) for inactivated coronavirus, Zika virus antigen, respiratory syncytial virus, virus subunits, and other infectious disease antigens [16,17,18,19]. PLGA has been used as a synthetic polymer in particle engineering and delivery systems due to its biocompatibility, biodegradability, and sustained release properties. Its safety profile has also been well established, earning approval from the U.S. FDA, where it is currently listed as a pharmaceutical excipient [20]. In terms of the method of formulating these particles, the double-emulsion formulation has the advantage of preserving the biological activity of the protein, as in the single-emulsification technique, the hydrophilic agents such as proteins or peptides can easily diffuse in the aqueous phase [21]. Moreover, there has been vast knowledge about the incorporation of adjuvants in vaccine formulation and development to boost the immune response [22]. AddaVax^TM^ (MF59-like), a squalene-based nanoemulsion, is an example of an approved adjuvant and has been demonstrated to enhance the immune response [23]. It works by inducing chemokines and cytokines that are crucial for the recruitment, activation, and maturation of antigen-presenting cells [24].

In terms of delivery systems, microneedles have gained attention as a delivery platform for antigens. They are tiny projection needles that are disrupted by force when applied to the skin and can mediate the release of either drug or antigen to the dermis or epidermis layer of the skin [25]. These needles are long enough to penetrate the epidermal and dermal layers of the skin. However, they are not long enough to activate the pain nerve endings. The different types of microneedles that can be fabricated include hollow microneedles, solid microneedles, and dissolving microneedles [26]. Importantly, dissolving microneedles is the most appropriate method in the transdermal delivery of vaccines because of their versatility in loading different types of antigens. Also, microparticulate-based antigens can be loaded onto them and can facilitate their release from their vehicles. A randomized clinical trial has demonstrated the safety, acceptability, and immunogenicity of an inactivated influenza vaccine delivered via microneedles [27]. However, this study also utilizes a microparticulate-based vaccine, which offers additional advantages such as enhanced antigen stability, controlled release, and improved immune responses. Further research into microparticulate formulations and dissolving microneedle systems could optimize vaccine delivery and efficacy.

Microneedles (MNs) facilitate transdermal vaccine delivery by disrupting the stratum corneum, allowing the passive diffusion of vaccine macromolecules. However, this approach is often limited by low delivery efficiency. In contrast, iontophoresis actively enhances transdermal transport but remains ineffective for vaccine macromolecules due to the formidable barrier of the stratum corneum [28]. Interestingly, Zheng et al. demonstrated that the synergistic combination of microneedle puncture and iontophoresis significantly enhances transdermal vaccine delivery. This approach creates microchannels that enable passive diffusion while simultaneously employing iontophoresis to drive vaccine molecules into the epidermis and dermis. These antigens are subsequently captured by antigen-presenting cells, leading to immune activation. This study utilized polyacrylamide/chitosan hydrogels, selected for their biocompatibility, conductivity, elasticity, and high loading capacity, as the primary vaccine storage matrix for iontophoresis-mediated delivery. The proposed “press-and-poke” strategy, incorporating microneedle-mediated penetration followed by iontophoresis-driven transport, demonstrated substantial improvements in vaccine delivery efficiency [29]. Despite these advancements, biosafety concerns and the need for stable conductivity between the electrodes and skin during iontophoresis remain challenges that require further investigation [30]. Additionally, modified MN patches fabricated from linezolid, boronated chitosan, polyvinyl alcohol, and D-sorbitol were engineered using a vacuum micromoulding method. The linezolid-loaded patches–iontophoresis combination eradicated biofilms and promoted the healing of infected oral mucosal wounds [31].

Beyond iontophoresis-driven microneedle patches, Straeten et al. reported an automated microneedle patch printing system for COVID-19 mRNA vaccines, housed within a standalone device. This platform utilizes a vaccine ink formulation composed of lipid nanoparticles loaded with mRNA and a dissolvable polymer blend, optimized for high bioactivity through in vitro screening. Preclinical studies in mice revealed that immunization via manually produced microneedle patches encoding the SARS-CoV-2 spike protein receptor-binding domain elicited long-term immune responses comparable to intramuscular administration, underscoring its potential as a viable vaccine delivery strategy [32]. Additionally, emerging research has focused on the development of polymer-based transdermal drug delivery systems fabricated using additive manufacturing technologies. One such system integrates a 3D-printed polymer-based micropump with a drug reservoir, coupled with a hollow microneedle array fabricated via photolithography. This design facilitates precise, low-power drug delivery against the backpressure of the skin, ensuring biocompatibility, stability, and high reliability [33]. The ability to fabricate these systems using scalable additive manufacturing technologies highlights their potential for personalized medicine and broad clinical applications in vaccine delivery.

While microneedle technology has significantly advanced transdermal drug and protein delivery, its application in controlled antigen release remains limited. Achieving a sustained antigen release from a microparticle depot in the skin after transdermal vaccination presents a powerful strategy to prolong antigen interaction with dermal dendritic and Langerhans cells, enhancing the immune response to the influenza vaccine. Thus, the integration of antigen-loaded polymeric microparticles with fast-dissolving microneedles represents an innovative platform for sustained antigen release and targeted transdermal vaccine delivery. From a manufacturing standpoint, conventional microneedle patch fabrication typically involves solvent casting, where the vaccine antigen is incorporated into a mold and allowed to dry, often assisted by vacuum or heat. Lyophilization, however, is generally unsuitable as it results in mechanically weak and porous structures due to void formation during sublimation [34]. Interestingly, despite the potential advantages, dissolving microneedles as a delivery system for microparticle-based influenza vaccines (H1N1 and H3N2) remains unexplored. The development of H1N1- and H3N2-loaded polymeric microparticles with fast-dissolving microneedles is an area that has yet to be fully investigated. Toward this effort, we have already optimized and developed dissolving microneedle formulations with a lyophilized model antigen [35].

Previous studies have demonstrated the use of whole inactivated influenza virus and virus-like particles in the development of a vaccine against the influenza A virus [23,36,37]. However, most of the vaccines are administered via the invasive intramuscular route. To advance this field, the transdermal route of vaccination with a microparticle-based vaccine is an attractive strategy that is being explored by our group. In a proof-of-concept study, we have previously demonstrated that the matrix-2 protein virus-like particle (VLP) vaccine administered via the transdermal route induced immunity against influenza A virus in a murine model [38]. Also, our research group has demonstrated immune induction against the influenza A virus by utilizing matrix-2 protein virus-like particles as an antigen, delivered through microneedles, with PLGA serving as the polymeric nanoparticle matrix [18].

In this current study, the inactivated influenza A H1N1 virus and inactivated influenza A H3N2 virus subtypes were encapsulated in a biodegradable poly (lactic-co-glycolic acid) (PLGA) polymeric microparticulate matrix, lyophilized, adjuvanted, and loaded onto dissolving microneedles. Next, microparticles were characterized, and the immunogenicity and cell cytotoxicity were characterized in cell-based assays. In vivo, we evaluated the antigen-specific induction of humoral, mucosal, and cellular response against both of the influenza A virus subtypes (influenza A H1N1 and inactivated influenza A H3N2) mediated by antigen-loaded PLGA microparticles using dissolving microneedles as a delivery system.

## 2. Materials and Methods

### 2.1. Materials

Sodium hyaluronate (100 kDa) was procured from Lifecore Biomedical (Chaska, MN, USA). The poly (lactic-co-glycolic acid) was obtained from Evonik Industries (North, Rhine-Westphalia, Essen, Germany), in a 75:25 ratio. Trehalose dihydrate (98.5% purity, HPLC grade), dichloromethane (99.5% purity, HPLC grade), DMSO (99.9% purity, HPLC grade), polyvinyl alcohol (PVA) (Avg Mol Wt. 30,000–70,000), and lipopolysaccharides (LPSs) from Escherichia coli O111:B4 were procured from Sigma-Aldrich (St. Louis, MO, USA). Influenza A virus, A/Scotland/1974, (H3N2), BPL-Inactivated, NR-49453 and influenza A virus, A/California/04/2009 (H1N1) pdm09, BPL-Inactivated, and NR-49450 antigens were provided by BEI Resources (NIAD, NIH; Influenza Virus) which they recommended. AddaVax^TM^ was purchased from InvivoGen (San Diego, CA, USA). Pierce Micro BCATM Assay Kit was purchased from ThermoFisher Scientific (Waltham, MA, USA). The 8 × 8 array polydimethylsiloxane (PDMS) MN templates were acquired from Micropoint Technologies (Singapore). Non-essential amino acids, Dulbecco’s Modified Eagle’s Medium (DMEM), fetal bovine serum (FBS), and penicillin/streptomycin were purchased from American Type Culture Collection (ATCC) (Manassas, VA, USA). Kenneth L. Rock of the Dana-Farber Cancer Institute, Inc. (Boston, MA, USA) donated murine dendritic cells (DCs). Swiss Webster mice that were six to eight weeks old were purchased from Charles River Laboratories (Wilmington, MA, USA). IgG, IgA, IgG1, and IgG2a secondary goat anti-mouse antibodies with HRP tags were purchased from Invitrogen (Rockford, IL, USA). Anti-mouse CD4 antibodies with allophycocyanin (APC) labeling and anti-mouse CD8 antibodies with fluorescein isothiocyanate (FITC) labeling were purchased from Invitrogen, Thermofisher Scientific (Waltham, MA, USA). All solvents used were HPLC grade.

### 2.2. Methods

#### 2.2.1. Formulation and Characterization of Inactivated Influenza A H1N1 and Inactivated Influenza A H3N2 Microparticulate-Based Vaccine

The inactivated influenza A H1N1 virus and inactivated influenza A H3N2 microparticulate vaccine were formulated by the double-emulsion and solvent evaporation method as previously reported [19]. In brief, each of the antigens (1.45% loading) was dissolved in a PH 7.4 phosphate buffer and added to PLGA dissolved in dichloromethane (DCM). The mixture was subjected to probe homogenization at 17,000 RPM for two minutes to form the *w*/*o* primary emulsion. Further, another cycle of probe homogenization was completed after the addition of a 0.1% PVA solution (continuous phase) to form the *w*/*o*/*w* double emulsion. Next, the residual acetone was removed by stirring the double emulsion at 500 RPM for 5 h at room temperature. The microparticle was washed by centrifugation at 15,000 RPM for 15 min at 4 °C to remove the excess PVA and resuspended with 1 mL of 2% trehalose solution. The formulation was pre-frozen for 2–3 h and lyophilized. After formulation, the size, surface charge, encapsulation efficiency, and shape of the microparticles were assessed under the scanning electron microscope and Malvern Zetasizer Nano ZS (Malvern Panalytical Ltd., Worcestershire, UK), respectively, as previously reported [39]. For encapsulation efficiency, here, to dissolve the PLGA matrix, 2 mg of the vaccine microparticle was suspended in dichloromethane (DCM). To extract the antigen, the suspension was centrifuged at 3000 RPM for 10 min at 25 °C to form a pellet. The concentrated antigen pellet was put in the hood vacuum for 30 min to evaporate the remaining DCM after the supernatant was removed. According to the manufacturer’s recommendations, the antigen was resuspended in 1 mL of PBS and subjected to a micro-bicinchoninic acid (BCA) test for analysis. The following formula was used to determine the percentage encapsulation efficiency (%EE) [40].

$$\%\;Encapsulation\;Efficiency=\frac{Experimental\;concentration\;of\;antigen\;in\;2mg\;of\;MP}{Theeoretical\;concentration\;of\;antigen\;in\;2mg\;of\;MP*100}$$

#### 2.2.2. In Vitro Determination of the Immunogenicity of Vaccine Microparticles

The microparticulate vaccine formulations were then tested for immunogenicity in an in vitro system. Nitric oxide (NO) and its metabolites nitrite and nitrate are produced by APCs like macrophages and dendritic cells in response to any external pathogen, and they are crucial for non-specific innate immunity [41]. Here, using Griess’s test, an increase in nitrite secretion was evaluated as a sign of immunogenicity. Specifically, murine DCs were plated at a density of 10,000 cells per well and pulsed with a calculated dose of antigen. Next, the cells were then incubated at 37 °C for 24 h. The group for this investigation includes 2 µg of lipopolysaccharide (LPS) (positive control), cell only (negative control), H1N1 MP (2 µg), H3N2 MP (2 µg), and (H1N1 + H3N2) MP. After 24 h of incubation, the supernatants (50 µL/well) were transferred to a new 96-well plate. Each well was then filled with 50 µL of 1% sulfanilamide in 5% phosphoric acid, which was then left to incubate for 5–10 min at ambient temperature while being shielded from light. To each well, 50 µL of 0.1% NED (N-1-naphthyl ethylenediamine dihydrochloride) in deionized water was added and then incubated for 5–10 min at room temperature. The presence of nitrite is indicated by the emergence of a purple or magenta color. A BioTek Synergy H1 plate reader (BIO-TEK Instruments, Winooski, VT, USA) was used to read the absorbance at 540 nm. A sodium nitrite standard curve was used to measure the nitrite concentration.

#### 2.2.3. Determination of Cytotoxicity of the Vaccine Microparticles Using MTT (3-(4,5-Dimethylthiazol-2-yl)-2,5-Diphenyltetrazolium Bromide Assay

To determine whether vaccine microparticles are not toxic to cells, the MTT assay was conducted. Here, 10,000 dendritic cells were seeded in each well of a 96-well plate. After this, the cells were subjected to a variety of treatments at different concentrations of H1N1 and H3N2 microparticles ranging from 31.25 to 1000 µg/mL, including cells only (no treatment) and 100 µL of dimethyl sulfoxide (DMSO; Sigma Aldrich, St. Louis, MO, USA). In total, 100 µL of the microparticle suspension was added to the corresponding wells in triplicate. Next, the cells were incubated at 37 °C and exposed for 24 h for cytotoxicity. After the incubation period, the wells were washed and treated with 10 µL of 0.5% *w*/*v* MTT reagent stock from Sigma Aldrich in St. Louis, Missouri, USA, and made up to 100 µL in volume with media. After that, the plate was incubated at 37 °C for 4 h to allow metabolically active (living) cells to produce the purple precipitate. To dissolve the precipitate, 100 µL of DMSO was added, and the plate was then wrapped in foil and kept on the shaker for 15 min. At 570 nm, the plate was read. Cell viability was calculated using the average absorbance of the cells-only group (untreated).

#### 2.2.4. Formulation of Dissolving Microneedles Containing Microparticulate-Based Vaccine

The dissolving microneedle containing the microparticulate-based vaccine was fabricated by the spin-casting method as previously reported [17]. Briefly, the microneedle gel base consists of 5% trehalose solution and 10% sodium hyaluronate. The required amount of microparticles equivalent to 20 µg of antigen was weighed and suspended in the trehalose solution and was mixed thoroughly after the addition of sodium hyaluronate. Finally, the pre-weighted PDMS MN mold, 25 mg of gel base, was subjected to centrifugation at 4000 RPM for 15 min at 15 °C and dried in a desiccator overnight. The following morning, a backing layer consisting of 10% HA solution was added.

#### 2.2.5. Dosing Schedule and Mice Vaccination Procedure

**Figure 1:**
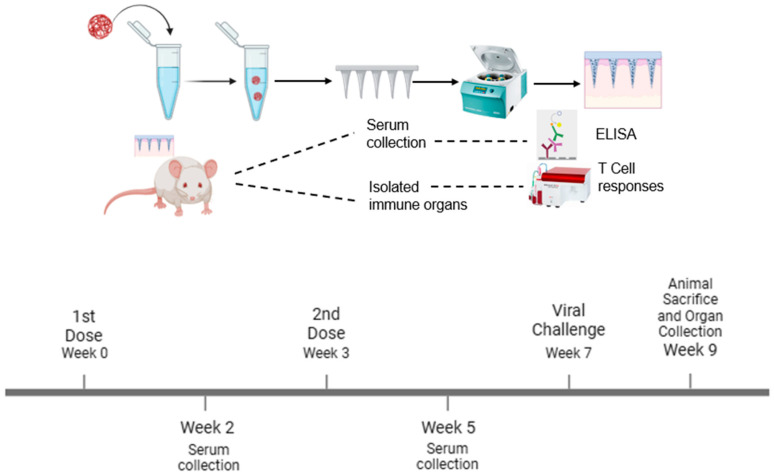
Schematic of the experiment. The vaccine microparticles were formulated and loaded into dissolving microneedles using the spin-casting method. The vaccine microparticles were administered using transdermal delivery with a one prime and one booster dose vaccination strategy. The serum antibody levels were quantified using ELISA, and T cell responses were determined using flow cytometry.

Using Swiss Webster (CFW) male mice (n = 5), aged 6 to 8 weeks, the vaccine’s immune response was evaluated in vivo using dissolving MN as follows: naïve group (no treatment), Fluzone group (3.0 µg administered via the intramuscular group), and the microparticle containing the equivalent of [H1N1(20 µg) + H3N2(20 µg)+ AddaVaxTM MF59 (10 µg)]-loaded MN patches group. The investigation was performed as per the approved Mercer University IACUC protocol no: A2404002. The microneedle groups were vaccinated via the dorsal region of the mice’s skin using an applicator, and the Fluzone group was vaccinated intramuscularly, which is the marketed control. For the MN administration, a 2 × 2 cm patch of fur was removed from the dorsal region after the mice were sedated (inhalational chamber using isoflurane) before vaccination using a depilatory cream one day before the vaccination. We used a one prime and a one booster dose vaccination strategy at weeks 0 and 3 as shown in Figure 1. To measure the antibody levels, the mice were bled from the tail at week 2 and week 5. At week 7, mice were intranasally challenged with the dual live virus H1N1(1 × 10^4^ pfu/mL) and H3N2 (50 µL of 5 × LD50 of A/PHILIPPINES/2/82) and observed for 2 weeks. At week 9, the animals were sacrificed, and their immune organs, including the spleen and lymph node, were separated and prepared into single-cell suspensions to examine T cell responses.

#### 2.2.6. Evaluation of Humoral Immune Response Using ELISA (Enzyme-Linked Immunosorbent Assay)

After each dose, serum samples were collected from the mice, and an enzyme-linked immunosorbent assay was used to determine the antigen-specific antibody levels for each of the influenza subtypes. Briefly, using high-binding 96-well plates, 0.2 µg/well of antigen in a carbonate buffer solution (PH 9.6) was coated and incubated overnight at 4 °C. Before blocking the plates with 3% Bovine Serum Albumin (BSA) in TPBS and incubating for 3 h at 37 °C, the wells were washed three times with 200 µL of 0.01% TPBS. Next, the plates were re-washed, and the diluted serum samples (50 µL/well) were added and incubated at 4 °C overnight. The next day, the plates were washed with TPBS, and horseradish peroxidase (HRP)-linked goat anti-mouse IgG, IgA, IgG1, and IgG2 antibodies (1:2000 to 1:4000) were added to the well plates and incubated for 1.5 h. After this, the plates were re-washed, before 50 µL of TMB(3,3,5,5-Tetramethylbenzidine) was added to each well and kept on the shaker for 10 min at room temperature. Finally, 50 µL of 0.3M H_2_SO_4_ was added to each well, and the absorbance at 450 nm was recorded using a plate reader. After sacrificing the mice, the same procedure was used for lung supernatants using a 1:2000 dilution for HRP-linked IgA and IgG.

#### 2.2.7. Analysis of Specific T Cell Responses in Lymph Node and Spleen

Following the sacrifice of the mice at week 9, the lymph nodes, both brachial and inguinal, and the spleen of the mice were collected and processed into a single-cell suspension using a 40 µm cell strainer. Here, the red blood cells were treated with ammonium chloride potassium (ACK) lysis buffer for three minutes and centrifuged at 1200 rpm for 10 min to remove the lysed RBCs. After centrifuging the cells at 1200 rpm, they were reconstituted in DMEM containing 70% fetal bovine serum (FBS) and 5% volume-to-volume DMSO and then frozen at −80 °C. A flow cytometer was used to assess the lymph nodes and spleen cells’ percentage (%) expression of CD4+ and CD8+ T cells. To remove the medium and DMSO, the cell suspensions were first immediately defrosted and centrifuged at 1200 rpm. DMEM was used to resuspend the cells, and 5 ng/mL of IL-2 was added overnight. The next day, the cells were resuspended in DMEM after being centrifuged at 1200 rpm to eliminate the IL-2. After that, the cells were stimulated overnight with 5 µg/mL of the inactivated specific antigen. The splenocytes and lymphocytes were activated in vitro with the vaccination antigen to determine the CD4+ and CD8+ T cell responses toward the influenza subtypes. The cells were centrifuged at 1200 rpm to create a pellet after being exposed to the antigen for a 24 h incubation period. The cells were resuspended in 100 µL of the marker solution, which is composed of anti-mouse CD4 and anti-mouse CD8 antibodies that have been APC and FITC tagged. The cells were placed on ice for an hour of incubation, kept out of the light, and gently vortexed every 15 min. The cells were incubated and then washed three times before being subjected to flow cytometric analysis.

#### 2.2.8. Statistical Analysis and Determination of Mice Sample Size

Each of the experiments was conducted in triplicate unless otherwise stated. We used GraphPad Prism 9.4.1 software (GraphPad Software, San Diego, CA, USA) for statistical analysis. We used one-way ANOVA and two-way ANOVA to measure normally distributed data with independent groups and dependent groups, respectively. A post hoc test was conducted to analyze multiple comparisons among groups. The data are presented as Mean ± SEM, with statistical significance defined as *p* < 0.05.

## 3. Results

### 3.1. Characterization of Vaccine Microparticles and Dissolving Microneedles Characterization

**Figure 2:**
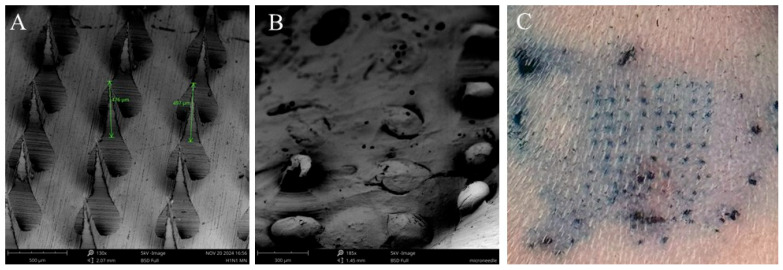
Characterization of vaccine microparticles and dissolving microneedles using scanning electron microscope. (**A**) Height of microneedles was 486 µm imaged at a magnification of 130×. (**B**) Dissolving microneedles after administration onto the skin of the mice for 10 min, imaged at a magnification of 185×, (**C**) Dissolving microneedles form pores on the skin as determined using methylene staining.

The sizes of the H1N1 and H3N2 vaccine microparticles were approximately 1470 nm and 1413 nm, respectively. The polydispersity index for H1N1 vaccine microparticles and H3N2 vaccine microparticles was 0.4, while the Zeta potential was found to be −26.4 mV and −23.8 mV, respectively, as shown in Table 1. The negatively charged particles show that they are stable, and a polydispersity index of less than 1 indicates that they are uniformly distributed. In terms of encapsulation efficiency, for H1N1 vaccine microparticles, it was 92%. For H3N2 vaccine microparticles, it was 91%, demonstrating that the double-emulsion method effectively facilitates high antigen loading, making it a reliable approach for vaccine formulation. When the dissolving microneedles were observed under scanning electron microscopy, they were very shaped needles with approximately 486 µm in length (Figure 2A). After administration, the SEM images also showed that the microneedles dissolved completely and formed pores on the skin of the mice after 10 min, as shown in Figure 2B,C, during methylene blue staining. For negatively charged microparticles with a Zeta potential below −30 mV, these properties ensure long-term stability, uniform dispersion, and efficient cellular uptake, which are essential for controlled drug release and vaccine delivery systems. Also, a polydispersity index of 0.4 indicates a homogeneous formulation with uniform particle size, leading to consistent drug release and predictable high-quality formulation.

### 3.2. Vaccine Microparticles Induce Immunogenicity as Evidenced by Nitric Oxide Release in Dendritic Cells

**Figure 3:**
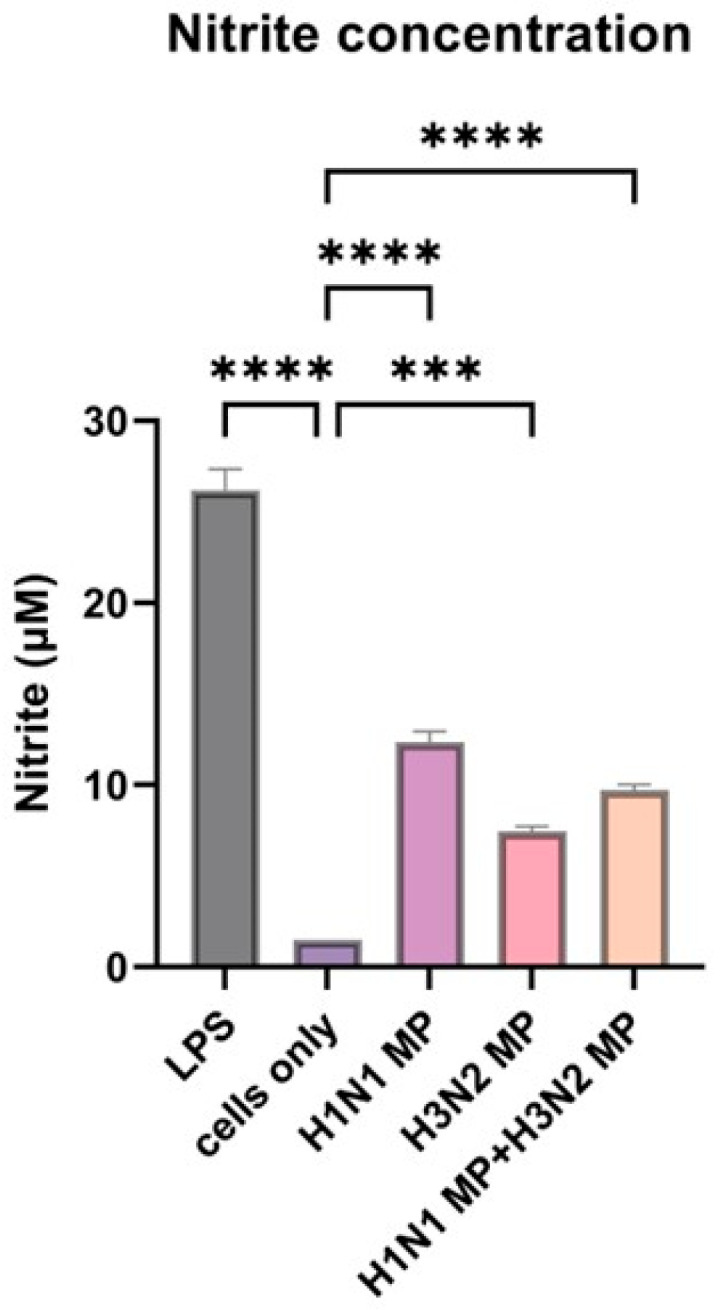
Nitrite oxide release from dendritic cells when pulsed with vaccine microparticles. The vaccine microparticles (MPs) induce a significant amount of nitrite oxide release. The release of nitrite oxide was assessed by the Griess assay in the supernatant. The cells were treated with the following groups: lipopolysaccharide (LPS), H1N1 microparticles, H3N2 microparticles, and H1N1 + H3N2 microparticles. Data expressed as Mean ± SEM n = 3, one-way ANOVA test, and post hoc Dunnett’s test were used for multiple comparisons. ns: non-significant, *** *p* ≤ 0.001, and **** *p* ≤ 0.0001.

To evaluate the ability of the vaccine-loaded microparticles to induce an immune response as evidenced by nitric oxide (NO) release, we performed a Griess assay. Nitric oxide secretion by dendritic cells serves as a key indicator of innate immune activation and can act as a precursor for stimulating a broader immune response. All cells treated with vaccine microparticles, including the combined form (H1N1 + H3N2) microparticles, induce significant nitrite oxide release (*p* ≤ 0.0001) compared to the cells that were not treated with the microparticles, as shown in Figure 3. The lipopolysaccharide (LPS) served as a positive control, and the untreated cells did not release significant nitric oxide.

### 3.3. Determination of Cytotoxicity of the Vaccine Microparticles Using the MTT Assay

**Figure 4:**
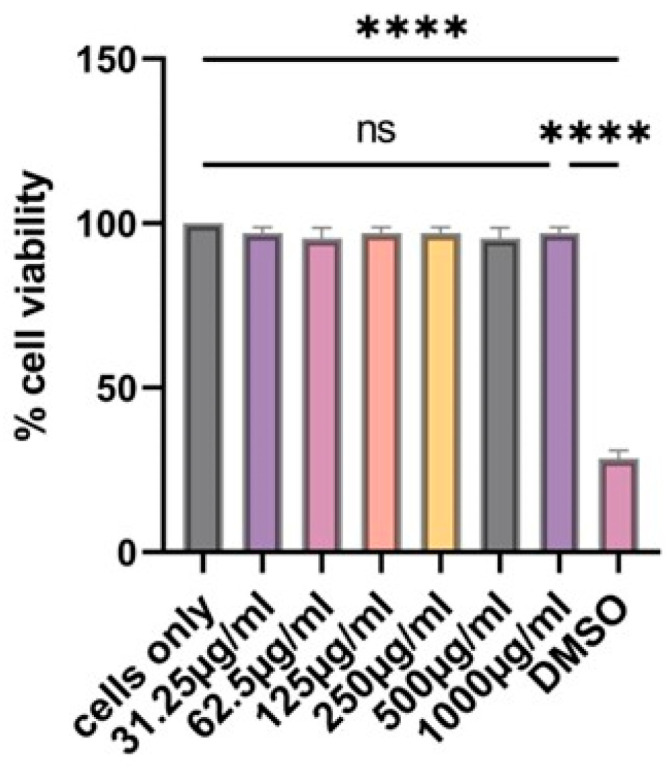
At 24 h, there is no statistically significant difference between the cells only and the cells treated with vaccine microparticles. Two-fold serial dilution of (H1N1 + H3N2) microparticles was performed in complete DMEM; the concentration range was 31.25–1000 µg/mL at a volume of 100 µL/well. Dimethylsulfoxide (DMSO) was used as a positive control. Data expressed as Mean ± SEM n = 3, one-way ANOVA test, and post hoc Dunnett’s test were used for multiple comparisons. ns: non-significant, and **** *p* ≤ 0.0001.

Using the MTT assay, we investigated if our vaccine microparticles are toxic to cells or not. In the past, we have tested the effect of different concentrations of microparticles on cells at 24 h. In agreement with what has been reported before, the particles are not toxic to dendritic cells for 24 h even at 1000 µg/mL as shown in Figure 4. Dimethyl sulfoxide (DMSO) was toxic to the cells, resulting in significant cell death, and was used as a positive control.

### 3.4. Vaccine Microparticles Induce High HIN1-Specific Serum Antibodies in Mice

**Figure 5:**
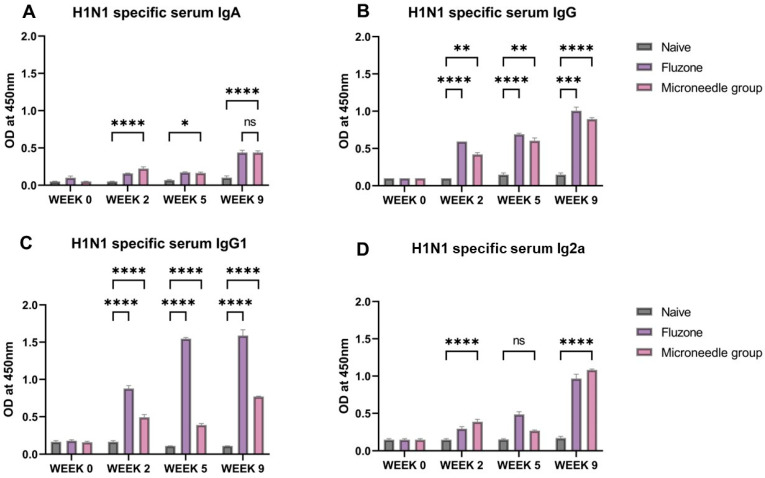
H1N1-specific serum antibody levels in vaccinated mice: (**A**) total serum IgG levels in vaccinated mice. (**B**) Total serum IgA levels in vaccinated mice. (**C**) IgG1 serum subtypes in vaccinated mice. (**D**) IgG2a subtypes in vaccinated mice. The responses obtained were compared to the naïve group, and Fluzone was used as a positive control. Data expressed as Mean ± SEM n = 5, one-way ANOVA test, and post hoc Dunnett’s test were used for multiple comparisons. ns: non-significant, * *p* ≤ 0.05, ** *p* ≤ 0.01, *** *p* ≤ 0.001, and **** *p* ≤ 0.0001. Fluzone is the intramuscular marketed control group (1.5 µg), and the microneedle group is H1N1(20 µg) + H3N2(20 µg).

The H1N1-specific IgA, IgG, IgG1, and IgG2a antibody levels were quantified at week 2, week 5, and week 9. The serum IgA levels were significantly higher (*p* ≤ 0.0001) at weeks 2 and 9. (Figure 5A). The serum IgG increased at week 2 and remained at this level until week 9. For the IgG subtypes, the IgG1 levels were increased and significant (*p* ≤ 0.0001) from week 2 until week 9 (Figure 5C). For IgG2a, there was a significant increase (*p* ≤ 0.0001) in the level at week 2, with a slight decline at week 5, but picked up again at week 9, as shown in Figure 5D.

### 3.5. Vaccine Microparticles Induce High H3N2-Specific Serum Antibodies in Mice

**Figure 6:**
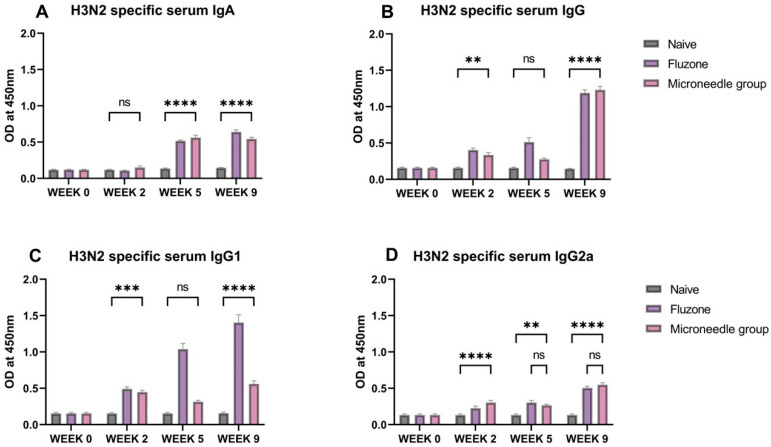
H3N2 specific serum antibody levels in vaccinated mice. (**A**) Total serum IgA levels in vaccinated mice. (**B**) Total serum IgG levels in vaccinated mice. (**C**) IgG1 serum subtypes in vaccinated mice. (**D**) IgG2a subtypes in vaccinated mice. The responses obtained were compared to the naïve group, and Fluzone was used as a positive control. Data expressed as Mean ± SEM n = 5, one-way ANOVA test, and post hoc Dunnett’s test were used for multiple comparisons. ns: non-significant, ** *p* ≤ 0.01, *** *p* ≤ 0.001, and **** *p* ≤ 0.0001. Fluzone is the intramuscular group (1.5 µg), and the microneedle group is H1N1(20 µg) + H3N2(20 µg).

In this case, the H3N2-specific IgA, IgG, IgG1, and IgG2a antibody levels were quantified at week 2, week 5, and week 9. The serum IgA levels were significantly higher (*p* ≤ 0.0001) at week 5 and week 9 (Figure 6A). A similar trend was observed for IgG1. The serum IgG was significantly higher (*p* ≤ 0.0001) at weeks 2 and 9 (Figure 6B). For the IgG subtypes, IgG1 levels were significantly higher (*p* ≤ 0.0001) at week 2 and week 9; however, for IgG2a, it increased from week 2 to week 9, as shown in Figure 6D).

### 3.6. Elevated Antigen-Specific Antibody Levels in Lung Supernatant Representing Excellent Mucosal Responses

**Figure 7:**
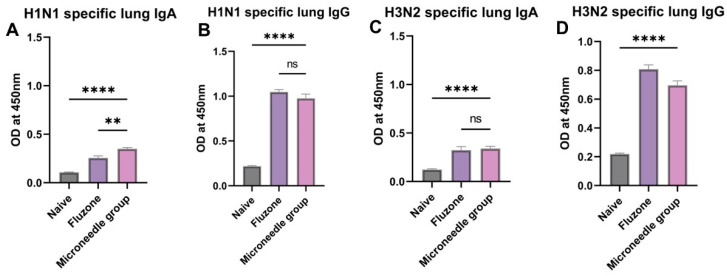
Antigen-specific lung antibody levels in vaccinated mice. (**A**) H1N1-specific lung supernatant IgA levels in vaccinated mice. (**B**) H1N1-specific lung supernatant IgG levels in vaccinated mice. (**C**) H3N2-specific lung supernatant IgA levels in vaccinated mice. (**D**) H3N2-specific lung supernatant IgG levels in vaccinated mice. The responses obtained were compared to the naïve group, and Fluzone was used as a positive control. Data expressed as Mean ± SEM n = 5, one-way ANOVA test, and post hoc Dunnett’s test were used for multiple comparisons. ns: non-significant, ** *p* ≤ 0.01, and **** *p* ≤ 0.0001. Fluzone is the intramuscular marketed control group (1.5 µg), and the microneedle group is H1N1(20 µg) + H3N2(20 µg).

We observed significantly high levels (*p* ≤ 0.0001) of IgA and IgG in the lung supernatant after sacrificing the vaccinated mice (Figure 7A–D). These antibodies are specific to H1N1 and H3N2. These higher levels were comparable to the marketed Fluzone vaccine that was administered through the intramuscular route.

### 3.7. Vaccine Microparticles Induce Cellular Responses in Mice

**Figure 8:**
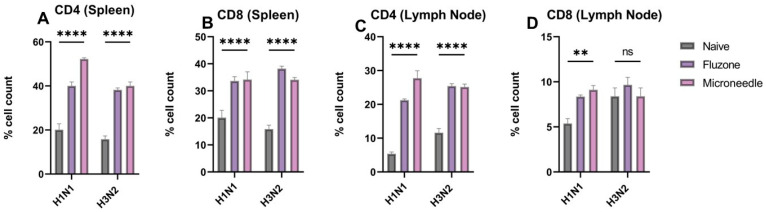
H1N1 and H3N2 cellular responses in vaccinated mice. (**A**) CD4 cells in the spleen of vaccinated mice. (**B**) CD8 cells in the spleen of vaccinated mice. (**C**) CD4 cells in the lymph node of vaccinated mice. (**D**) CD8 cells in the lymph node of vaccinated mice. The responses obtained are compared to the naïve group. Data expressed as Mean ± SEM n = 5 and two-way ANOVA test. ns: non-significant, ** *p* ≤ 0.01, and **** *p* ≤ 0.0001.

We observed significantly higher responses (*p* ≤ 0.0001) to the expression of CD4- and CD8-positive cells in the spleenocytes and the lymphocytes (Figure 8A–D). These responses are specific to H1N1 and H3N2. In the spleen, there was a 50% and 40% expression of CD4 cells, which are specific for H1N1 and H3N2, respectively. A similar expression of CD8 cells was observed. In the lymph node, there was a 27% and 25% expression of CD4 cells that is specific for H1N1 and H3N2, respectively. In terms of CD8 expression, they were significantly specific to H1N1. However, there was no difference between the microneedles group, the Fluzone group, and the naïve group specific to H3N2.

## 4. Discussion

In this study, we selected inactivated influenza A H1N1 and influenza A H3N2 viruses as influenza antigens due to their historical role in past pandemics and their future pandemic potential [3]. According to WHO surveillance data, these strains are among those predicted to circulate annually [42]. Although influenza viruses are highly prone to mutation, and the ultimate goal remains the development of a universal vaccine, the US Center for Disease Control (CDC) study indicates that even when vaccine components differ from circulating strains, they still provide a measurable level of protection within the population, such as the pediatrics [43]. This rationale supports our choice of antigens for this study. Furthermore, our approach aligns with previous research, as the same strains and subtypes were utilized in a randomized clinical trial, further reinforcing the relevance of our selection [27].

Next, we encapsulated these antigens in a biodegradable PLGA polymeric nanoparticle (75:25 ratio) and characterized them for size, polydispersity index, Zeta potential, and encapsulation efficiency. Particulate antigens and adjuvants offer a larger surface area with charged, hydrophobic, or receptor-interacting properties, facilitating better interaction with APCs compared to their soluble counterparts [44]. The efficiency of antigen uptake by antigen-presenting cells (APCs) is size-dependent. Particles smaller than 200 nm can directly enter lymphatic capillaries, facilitating rapid immune activation. In contrast, larger particles (ranging from 500 nm to several microns) are primarily transported to the spleen by APCs, influencing the nature and location of immune responses [45]. In this study, the formulated and lyophilized vaccine microparticles were approximately 1.4 µm in size, suggesting that they would likely be phagocytosed by APCs [13,14]. Once internalized, APCs migrate to lymphoid tissues, where the microparticles undergo processing within endosomes and are subsequently presented through the Major Histocompatibility Complex Pathways [45]. The surface charge of the vaccine microparticles is −26.4 mv (negatively charged) and aligned with previously reported findings [17,46]. In the preparation of polymeric nanoparticles, particle stability is maintained when microparticles exhibit a high positive or negative charge, preventing agglomeration and ensuring uniform dispersion, and the presence of carboxylic subunits impacts the negative charge of PLGA [38]. The encapsulation efficiency of the inactivated influenza antigen in PLGA MPs was >90%, demonstrating that the double-emulsion method effectively facilitates high antigen loading, making it a reliable approach for vaccine formulation.

We investigated the immunogenicity of vaccine microparticles by quantifying nitric oxide release using the Griess assay. Nitric oxide is an innate immune marker released by antigen-presenting cells upon exposure to foreign pathogens. Its release can recruit additional antigen-presenting cells to combat invading pathogens. Consistent with previous reports, we found particulate vaccines to be more immunogenic and capable of stimulating dendritic cells more effectively [19,27,47]. Demonstrating the therapeutic feasibility of our vaccine microparticles, on the other hand, our in vitro cytotoxicity assay revealed that our vaccine microparticles are not toxic to cells at the tested concentrations. Even when they were combined, (H1N1 + H3N2) Microparticles were not toxic to cells. To further strengthen the immune response and immunogenicity of our vaccine microparticles, we added AddaVaxTM (MF-59) as an adjuvant in the formulation, which helps in the maturation and recruitment of immune cells to the site of infection. In this study, we chose AddaVaxTM (MF-59) as our adjuvant because it enhances T cell activation and antibody responses, particularly in elderly and high-risk populations, and induces both Th1 and Th2 responses. We previously demonstrated that the influenza ectodomain matrix protein encapsulated in PLGA nanoparticles exhibits an initial burst release followed by a sustained release profile, with a release half-life of approximately 24 h in PBS [18].

Additionally, we investigated the immune response induced by vaccine microparticles administered in mice using dissolving microneedles (n = 5). The purpose of using fast-dissolving microneedles is to utilize biocompatible and water-soluble materials, such as sugars, to produce needles that completely dissolve in the skin, leaving no biohazardous sharps or tips after use. Hyaluronic acid was used as the polymer due to its high water-binding capacity, which enhances skin hydration. Dissolving microneedles, composed of hyaluronic acid, effectively soften and dissolve within biological tissues upon penetration. These microneedles dissolve within 10 min upon application, enabling rapid antigen uptake by antigen-presenting cells for a quick immune response. Dissolving microneedles minimizes the risk of tissue damage caused by mechanical forces during application, making them a safe and effective delivery system [48]. Furthermore, the literature reports indicate that the natural polymer gelatin has been used to formulate dissolving microneedle patches, demonstrating the immunogenicity of each component of the trivalent influenza vaccine (H1N1, H3N2, and B) in mice. Compared to the standard intramuscular injection recommended for human vaccination, this approach highlights the potential of transdermal delivery as an innovative and promising alternative [42]. Also, we have chosen the transdermal route of administration because the skin has specialized antigen-presenting cells, such as the dermal dendritic cells, macrophages, and Langerhans cells, which can process these antigens and present them to the cells of the adaptive immune system.

With this transdermal delivery system, our vaccine-induced higher antibody levels of IgG and IgA, which are specific to H1N1 and H3N2. A similar observation was reported in the published data [42]. To fully understand the type of Th-mediated immune response (Th1 and Th2) elicited by the vaccination response, we conducted an IgG subtyping analysis. A Th2-type response is typically associated with elevated IgG1 levels, which signal helper T cells to activate host immune defenses against invading pathogens. In contrast, a Th1-type response is linked to increased IgG2a levels, which stimulate cellular immunity by recruiting cytotoxic T cells and antigen-presenting cells to the site of infection [49,50]. Our findings showed that microneedle vaccination led to both Th1 and Th2-mediated responses. In previous studies, a two-booster dose regimen was used; however, in this study, we adopted a single prime and one booster dose vaccination strategy to achieve dose sparing. This approach not only reduces the number of booster doses required but also optimizes vaccine utilization. By minimizing the number of doses, this strategy enhances accessibility, making vaccination more efficient without compromising immunogenicity. By leveraging advancements in immunology, nanotechnology, and novel delivery systems, researchers can create more effective and scalable vaccines to combat emerging and re-emerging infectious diseases.

Given that the virus is transmitted via mucosal routes, it was essential to evaluate whether the vaccine could elicit an antibody response at the site of infection [51,52]. To assess the antibody levels in the lungs, we collected lung supernatants and analyzed IgA and IgG levels, which were significant. This finding underscores the potential of our vaccine to confer protective immunity at the primary site of viral entry, further supporting its effectiveness in preventing infection and disease progression.

In the research development of the influenza vaccine, there has been a focus on how increased CD4 and CD8 cell responses against conserved viral epitopes can confer heterosubtypic protection against influenza [53]. The percentages of CD4+ cells and CD8+ cells in the lymphocytes and splenocytes were analyzed using flow cytometry. The results show that the vaccine induced significant levels of cellular responses. Although CD4+ T cells were more abundant, the vaccine also elicited a heightened CD8+ T cell response. The significant induction of both CD4+ and CD8+ T cells highlights the vaccine’s ability to generate a balanced immune response, which is essential for effective protection.

The incorporation of particulate systems, such as PLGA microparticles, into dissolving microneedles has been explored to enhance immune responses and conserve all epitopes compared to dissolving microneedles without such systems. For example, it has been previously shown that an adjuvanted SARS-CoV-2 spike protein-based microparticulate vaccine, delivered by dissolving microneedles, induces significantly higher levels of antibodies in both serum and lung homogenates compared to the group that received spike RBD suspension in microneedles [54]. Separately, a study investigated the T cell responses induced by dissolving microneedles loaded with PLGA microparticles containing an antigen, comparing them to responses from intradermally injected aqueous PLGA microparticle formulations. The findings suggested that the inclusion of PLGA microparticles in dissolving microneedles could potentially enhance the immune response against the antigen [55,56]. However, it is important to note that dissolving microneedles without particulate systems has also induced immune responses.

Mazara et al. explored the development of controlled-release microparticles made of poly (lactic-co-glycolic acid) (PLGA) that stably encapsulate various antigens through aqueous active self-healing encapsulation (ASE) [57]. These microparticles are incorporated into rapid-dissolving microneedle patches for intradermal vaccination. PLGA microparticles containing Alhydrogel are loaded with antigens separate from microparticle fabrication using ASE. This avoids antigen exposure to many stressors. The microparticles demonstrate bi-phasic release, with an initial burst of soluble antigen, followed by delayed release. In animal models, these patches generate robust immune responses that are comparable to conventional intramuscular administration techniques [57]. This lays the framework for a versatile vaccine delivery system that could be self-applied with important logistical advantages over hypodermic injections.

The integration of iontophoresis-assisted dissolving microneedles with active self-healing encapsulation represents a transformative approach in transdermal vaccine delivery, offering precise control over antigen release kinetics, whether for timely burst release or sustained long-term delivery. By embedding antigen-loaded PLGA microparticles within, release profiles can be fine-tuned based on polymer degradation rates, while iontophoresis provides real-time modulation, allowing for on-demand vaccine release based on immune response needs. This could be further enhanced by smart skin-embedded systems, integrating biosensors to monitor immune markers and dynamically adjust antigen release via external stimuli like electrical pulses.

In future studies, we will examine and characterize long-term immunity mediated by this delivery system by determining the memory responses by analyzing memory markers in isolated B cells and T cells, such as CD45R and CD62L.

## 5. Conclusions

PLGA microparticles encapsulating inactivated viruses demonstrated strong immunogenicity, eliciting antigen-specific humoral responses such as IgA, IgG, IgG1, and IgG2A antibodies. Additionally, vaccine microparticles formulated with adjuvants significantly induced cellular responses, as well as the CD4+ and CD8+ T cell responses in immunized mice. These findings highlight the potential of incorporating inactivated viruses into a biodegradable PLGA polymeric matrix with adjuvants, delivered non-invasively via a fast-dissolving microneedle system, as an innovative approach for influenza A vaccination. The ease of transdermal vaccine administration makes it a promising strategy for mass immunization during pandemics, supporting its further development as a scalable and effective vaccine platform.

## Figures and Tables

**Table 1:** Characterization of vaccine microparticles.

Properties	H1N1 MP	H3N2 MP
Size	1470 nm ± 108.77	1413 nm ± 117.1
Polydispersity Index	0.4 ± 0.1	0.4 ± 0.1
Zeta Potential	−26.4 mv ± 4.609	−23.8 mv ± 3.5
Encapsulation Efficiency	92% ± 5%	91% ± 5%

## Data Availability

The original contributions presented in this study are included in the article.
